# Microbial water quality investigation through flow cytometry fingerprinting: from source to tap

**DOI:** 10.1093/sumbio/qvae003

**Published:** 2024-02-14

**Authors:** Leila Claveau, Neil Hudson, Peter Jarvis, Paul Jeffrey, Francis Hassard

**Affiliations:** Cranfield Water Science Institute, Cranfield University, College Road, Cranfield, Bedfordshire MK43 0AL, United Kingdom; South East Water, Rocfort Road, Snodland, Kent ME6 5AH, United Kingdom; Cranfield Water Science Institute, Cranfield University, College Road, Cranfield, Bedfordshire MK43 0AL, United Kingdom; Cranfield Water Science Institute, Cranfield University, College Road, Cranfield, Bedfordshire MK43 0AL, United Kingdom; Cranfield Water Science Institute, Cranfield University, College Road, Cranfield, Bedfordshire MK43 0AL, United Kingdom

**Keywords:** disinfection, flow cytometry, drinking water, water quality, fluorescent fingerprint analysis, faecal indicator organisms (FIO), *E. coli*

## Abstract

Ensuring the quality of treated drinking water is crucial for preventing potential health impacts, regulatory fines, and reputation damage. Traditional culture-based microbiological methods often fail to capture the heterogeneity of the bacterial communities in drinking water. This study employed daily interstage monitoring and flow cytometry (FCM) analysis over a period of one year to investigate the dynamics of water treatment processes and service reservoirs. The objective of this study was to test the utility of FCM fingerprints for aiding in microbial event detection. We found that the chlorine concentration contact time was pivotal for microbial log reduction across the treatment works. FCM fingerprints exhibited significant deviations during operational events, such as process interruptions, but did not correlate with the presence of bacterial indicator organisms in the distributed and tap water. Furthermore, the diversity of bacterial fingerprints, quantified by the Bray–Curtis dissimilarity index, served as an indicator for identifying potentially poor microbial water quality. In chlorinated waters with low cell counts, the background signal shows potential as a metric to differentiate between different water sources, thereby offering the possibility to characterize breakthrough events in these circumstances that challenge most other microbial analytical methods. Interestingly, groundwater from simpler treatment works showed a higher occurrence of bacterial indicators, whereas surface water works had a lower incidence. These findings underpin the importance of appropriate disinfection even for “low-risk” source waters and the added value that the statistical interpretation of FCM data can offer objective decision making.

**Sustainability Statement**

The paper's focus on enhancing treated drinking water quality is linked to multiple sustainable development goals (SDGs). It directly supports SDG 6 by using advanced methods, such as flow cytometry (FCM), to improve water treatment and distribution, which is essential for public health and well-being. Additionally, this research aligns with SDG 3 by aiming to prevent health issues from contaminated water, which is crucial for controlling waterborne diseases. The innovative approach of FCM and data interpretation also ties into SDG 9, advocating resilient infrastructure and innovation in water treatment technology. These advancements are the key to the development of sustainable and reliable water systems.

## Introduction

Ensuring the microbial safety of drinking water is of paramount importance for protecting public health. Microbial water safety is influenced by various factors, including source water quality, treatment processes used in water treatment works (WTWs), and integrity of the drinking water distribution system (DWDS) (Liu et al. 2013). However, contemporary challenges such as climate change, water scarcity, and polluted water sources complicate the consistent production of microbially ‘safe’ water, compromising public health protection and increasing costs to utilities and consumers(Semenza and Menne 2009). Traditional methods of assessing microbial quality, such as culturing bacterial indicators, provide only an indicative snapshot of pathways for faecal contamination, rather than a comprehensive hygienic assessment(Hassard et al. 2016). These culture-based tests often overlook a significant portion of bacteria (viable but non-culturable organisms) and do not easily distinguish between pathogenic and infectious organisms and less harmful ones(Pinto et al. 2015). Furthermore, culture-based quantification is time-consuming, with results often available too late to inform practical interventions regarding treatment, distribution, or water use (Van Nevel et al. 2017). Although advanced techniques, such as quantitative PCR assays and next-generation sequencing(Singhal et al. 2015, Farkas et al. 2017), offer detailed insights and more rapid data, they are not without challenges. These methods can struggle with the specificity and sensitivity necessary for identifying and quantifying viable pathogens or indicator organisms within complex environmental water samples, a crucial aspect of ensuring water safety and public health(Liu et al. 2017).

Within this framework, flow cytometry (FCM) is recognized as a transformative approach to assess microbial water quality, a method that has been widely embraced by the water sector in some countries. FCM has been applied for rapid, reproducible, and direct assessment of microbial populations in water (Hoefel et al. 2003, 2005). Applications range from operational assessments and root cause analysis to validation of water disinfection(Nocker et al. 2017), contributing significantly to our understanding of microbial dynamics across water treatment systems (Cheswick et al. 2019), and importantly, is amenable to automation for rapid user-free diagnostics (Berney et al. 2008). However, this technology is not without its limitations. FCM's wide-spectrum analysis presents challenges in identifying potential risks associated with specific pathogens of concern (Thom et al. 2022), such as viruses (Safford et al. 2023), and in differentiating them from the general microbial background, somewhat limiting its deployment for comprehensive and direct hygienic monitoring.

This study aims to extend the value of FCM in water quality management by employing fluorescent fingerprint analysis of water samples. This innovative approach, utilizing comprehensive fingerprinting that encompasses both cellular structures and their surrounding environmental background (debris and organic and inorganic particles), aims to discern phases of elevated risk across diverse water sources by detecting unusual microbial communities that signal potential contamination or microbial regrowth(Schleich et al. 2019). By correlating multivariate data extracted from FCM with potential changes in genotypic or phenotypic structures (Pluym et al. 2023), this research aims to enhance FCM's usefulness of through an ecological biodiversity metrics assessment (Props et al. 2016). Sophisticated and proven techniques developed for this purpose, such as cytometric histogram image comparison (CHIC) and FlowFP, which can elucidate the complexities of microbial communities in water, (De Roy et al. 2012, Koch et al. 2013) are applied here within the context of source-to-tap assessment of a full-scale WTW and DWDS. (Sadler et al. 2020).

This research navigates these scientific frontiers through a study that examines interstage datasets across WTWs and DWDs from 85 WTWs and 231 service reservoirs (SRs) in southern England over a year. This study investigates how different treatment processes and chlorine contact times (CTs) influence microbial water quality and how these factors correlate with different components of microbial fingerprints. Furthermore, the study explored FCM's latent ability of FCM to identify when bacterial regulatory compliance issues or public health incidents may occur within a specific WTW and its DWDS, presenting an opportunity for enhanced water safety management protocols. By integrating FCM and advanced data analysis techniques, this study offers unique insights into microbial compliance events, their possible causes, and associations with raw water quality and water treatment efficacy. It holds promise as a framework to significantly improve strategies to safeguard public health by ensuring the consistent production of microbially safe drinking water despite the growing environmental and operational challenges faced globally.

## Material and methods

### Sampling strategy

The study analysed water quality by examining samples from all 85 WTWs from a supply region in Southern England (UK). This provided an appropriate spatial resolution within a supply region that had similar operation practices, operating policies (for example, for disinfection), and control setups, permitting direct comparison. Side-by-side comparisons were carried out for sites with similar water source types and treatment approaches deployed. These WTWs process water from various sources: groundwater (GW), surface water (SW), or a blend of both, subsequently serving 231 SRs within different DWDSs. Distances between the WTWs and their respective SRs varied, with some having immediate post-treatment storage and others being located at a distance from the works (max 16 km). To assess the microbial log reduction credit, both raw and final water samples were drawn from 35 WTWs, which enabled a side-by-side comparison of their cell removal effectiveness. The treatment processes at these WTWs can be classified as simple disinfection by inline chlorination (*n* = 9), chlorine contact tank (CCT) (14), and more extensive flowsheets such as microfiltration (MF) membrane/CCT (4), rapid gravity filtration (RGF)/granular activated carbon (GAC)/CCT (3), and dissolved air flotation (DAF)/RGF/Ozone-GAC/CCT (2). Other sites have a more comprehensive treatment system because it is more challenging to treat water sources involving a clarifier (CF), RGF, ozone-GAC, UV, and CCT (3). The sampling frequency was weekly for WTW and monthly for DWDS samples, as this aligned with the regulatory monitoring requirements for water utility. Daily sampling was carried out at one WTW, which was under close inspection because of potential operational challenges impacting the water quality. The GW was predominantly treated by the first four flowsheets. The latter two processes are used to treat SW or blended supplies.

Sampling was conducted in triplicate at WTWs and SRs from April 2019 to March 2020. Raw, final, and SR outlet water samples were collected to ascertain how the WTW process performance and storage conditions influenced total cell counts (TCCs) and intact cell counts (ICC), as well as the potential for regrowth across the distribution network. Sub-populations of high nucleic acid content (HNA) and low nucleic acid content (LNA) bacteria were assessed using static secondary gating approaches validated in earlier studies (Claveau et al. 2024) that have been widely applied for the analysis of drinking water when using FCM (Prest et al. 2013). The study indirectly monitored potential regrowth/ingress by quantifying ICC within the DWDS, taking into consideration the impact of disinfection residuals and temperature as important factors governing cell populations. The influence of water storage was explored by comparing the hydraulically linked WTWs and their SR.

To assess the impact of operational events on the microbial population, one WTW with SW feed was chosen as a case study for higher-resolution interstage monitoring. This WTW (referred to as WTW F) was sampled daily for treated water, weekly for raw water, and at each stage of the treatment process. Two of its linked SRs (referred to as A and B) and tap water from its supply network were also included as they were exclusively sourced from this WTW during the study period. The microbial population was assessed by FCM fluorescent fingerprint analysis and enumeration using static gating for cell counts and phenotypic appraisal using FCM (method outlined below), with organic and inorganic backgrounds contributing to the analysis after the method outlined in earlier work (Claveau et al. 2024). Each sampling event was conducted in duplicate, following the regulatory sampling procedures outlined by Clark (2015). Specifically, the sample taps were flushed for 3 min, and flame-sterilized before sampling. Subsequently, taps were flushed for an additional 30 s before the collection of samples into sterile 250 ml sample bottles. These bottles contained a pre-aliquoted dose of sodium thiosulfate to reach 0.1% (w/v) in the water sample, which was sufficient to quench the residual chlorine. Upon collection, samples were transported to the laboratory in the dark for analysis within 24 h and were stored at temperatures between 4°C–8°C during transit.

### Water chemical, physical, and microbial parameters

The following physical and chemical constituents were measured using standard methods: turbidity (2130), pH (4500), and both total and free chlorine residual (4500). (APHA-AWWA-WEF 2012). Microbiological assessments were carried out on samples collected following standard methods derived from(APHA-AWWA-WEF 2012). *Escherichia coli* and total coliforms were analysed using the Colilert^®^ test (IDEXX, UK) based on the most probable number (MPN) enzyme substrate method (9223). This test uses a defined substrate technology, wherein bacterial enzymes react with specific nutrients, causing a colour change when the target bacteria, *E. coli* or coliforms, are present. A heterotrophic plate count (HPC, 9215) at 22°C (68–72 h) was used to determine the total viable count. *Clostridia perfringens* was measured using the membrane filtration method (9260), the water sample was filtered, the bacteria-retaining filter was placed on a selective medium and incubated at 44°C, and the appearance of black colonies after a 24-h incubation period indicated the presence of *Clostridia perfringens*. For these sites, log-reduction calculations were performed for TCC and ICC, and the results were stratified according to different WTW types, including appraisal of the removal across the entire treatment work (i.e. incorporating disinfection processes and upstream processes).

### Determining process performance

The free and total residual chlorine, reservoir/tank water levels, and flow rates were sourced from a database that received 15-min interval data from automated meter readings at various locations, including the WTW, DWDS, SR outlet, and final water. The chlorine concentration was ascertained using a number of online probes calibrated on a monthly basis.

In this study, we employed the concept of Chlorine CT, a critical parameter in water disinfection, representing the product of the concentration of chlorine (C, in mg/l), and the duration of water exposure (t, in minutes) (Rush 2002). The Ct is important as it quantifies the disinfection strength, informing the likelihood of microbial inactivation. Ct was approximated (Equation 1) using the minimum CCT volume and peak flow at each WTW, presenting the lowest potential exposure or worst-case scenario based on the free chlorine concentration.


(1) \begin{eqnarray*}
Ct = C{l}_{min}{\mathrm{\ }}\left( {mg.{L}^{ - 1}} \right) \times \frac{{{\mathrm{\ }}{V}_{min}{\mathrm{\ }}\left( {{m}^3} \right)}}{{{Q}_{max}{\mathrm{\ }}\left( {{m}^3/min} \right)}}
,
\end{eqnarray*}

where Cl_min_ the minimum recorded free chlorine concentration, V_min _= minimum volume of water in the contact tank, and Q_max _= maximum recorded flow rate at CCT.

CCTs are crucial for water disinfection, as they rely on ideal hydraulic performance and minimal short-circuiting. Effective design, regular flow assessments, and configuration adjustments are essential for preventing dead zones and ensuring efficient mixing and flow (Sobsey 1989). Seasonal temperature changes that affect the water viscosity and flow rates are also important considerations (Cheswick et al. 2022). This study primarily focused on nominal Ct but also considered the role of temperature and pH on disinfection efficacy (Hua and Reckhow 2008, Nocker et al. 2017). Lower pH and higher temperatures enhance chlorine disinfection, whereas alkaline conditions and cooler temperatures have the opposite effects. Additionally, these factors influence post-treatment microbial populations. Spatial analysis of the WTW outlets and SRs provided insights into chlorine residuals and overall Ct in the water supply zone.

### Fluorescent staining and flow cytometric measurements

Sample preparation commenced with dilution of samples exceeding TCC and ICC concentrations of 1 000 000 and 800 000 cells/ml, respectively, using 0.22 µm and 0.1 µm polyethersulfone (PES) filtered sterile Evian™ water. The samples were then stained for TCC and ICC. TCC staining involved the use of SYBR Green I (SGI, 10 000 × stock. S-7567; Thermo Fisher Scientific, UK), filtered with dimethyl sulfoxide (DMSO) (0.22 µm, Z290807, Sigma–Aldrich, UK), and diluted to a 100x working concentration. The ICC stain was prepared using a mixture of five parts of 100x SGI and one part of propidium Iodide (PI) (Thermo Fisher Scientific, UK). The final concentration of SYG was a 1 × and PI was 3.0 µM. Samples staining took stained in 96-well plates, which were then incubated using a microplate thermoshaker (Grant Instruments™ PHMP, Thermo Fisher Scientific, UK) at 35°C and 400 rpm for 15 min. Each sample (50 μl) was analysed at a flow rate of 66 µl min^−1^ with a BD Accuri C6 flow cytometer (Becton Dickinson UK Ltd., UK), employing a 488 nm solid state laser. Green and red fluorescence were measured at 533 nm (FL1) and 670 nm (FL3), respectively. A single static primary gate was applied to delineate bacteria from background signals and intact or total cells, following Prest et al. (2013). The threshold for the green fluorescence (FL1-H) channel was set at 480 arbitrary units (A.U). Microorganism numbers in this gate post-staining with SGI or SGI/PI formed the basis for TCC or ICC per ml calculations using a dynamic gating approach to account for differences in the observed microbial populations, especially following chlorination (Claveau et al. 2024). HNA and LNA content bacteria (HNA and LNA, respectively) were determined using a static secondary gate (Gatza et al. 2013, Prest et al. 2013). Log reductions across the WTW process steps were calculated after the determination of TCC and ICC concentrations, derived from average replicated measurements per sample using Equation 2.

Equation 2: Calculation of the log reduction of cells in each WTW.


(2) \begin{eqnarray*}
Log{\mathrm{\ }}\mathit{reduction} = Lo{g}_{10}\left( {\frac{{\mathit{Average}{\mathrm{\ }}\mathit{inlet}{\mathrm{\ }}\mathit{cells}{\mathrm{\ }}per{\mathrm{\ }}mL}}{{\mathit{Average}{\mathrm{\ }}\mathit{outlet}{\mathrm{\ }}\mathit{cells}{\mathrm{\ }}pel{\mathrm{\ }}mL}}} \right).
\end{eqnarray*}

### Data analysis

The data were processed using MS Excel, RStudio, and IBM SPSS Statistics version 22. Following the assessment of independence and normality, Welch's *t*-test was applied to determine the significance of the effects of various treatment technologies on TCC and ICC removal. Linear regression analysis was used to model the relationship between Ct values and ICC, providing insights into the impact of disinfectants on the microbial population.

### FCM fingerprint analysis

Fingerprint analysis was conducted using CHIC using FlowJo (v10.7.2), ImageJ (v1.54d-), and R (v2022-02-3) software (Koch et al. 2013, 2014). Briefly, FCM scatter plots were extracted using the BD Accuri C6 software. FCS files were converted to grey-scale pixel images using FlowJo. The ICC fingerprint was specifically utilized given its significance in assessing viability within chlorinated systems. Prior to the fingerprint analysis, neither inorganic nor organic backgrounds were removed. FCM scatterplots were converted into 300 × 300 pixel images with a 64-channel grey-scale resolution for image comparison. Comparisons of the cytometric images were conducted using the ImageJ software. The “generate disjunction” function produced an XOR image from two input cytometric images. The second algorithm produces an overlap, describing the pixel differences between the water samples. The average grey value per pixel was determined by calculating the sum of all pixel values from the XOR image and the number of pixels from the overlap image. This process represents a form of ‘phenotypic’ community analysis that is relevant to FCM fluorescence data (Pluym et al. 2023). Through this analysis, the FCM data were subjected to transformation, discretisation, and then concatenated into a single-dimensional vector, which was used as the basis for subsequent characterisation. Additional statistical measures, such as Bray-Curtis analysis and the construction of non-metric multidimensional scaling (nMDS) plots and clustering analysis, were performed using R.

During this procedure, a Bray-Curtis dissimilarity index was formulated to be presented as a matrix. The results were visualized using nMDS and correlation vector analysis by overlaying Pearson's correlation test on components within the nMDS. Microbial parameters (total viable count, coliforms, and *E. coli*) were assessed using the R function ‘envfit’ with the vegan package(Oksanen et al. 2007). Only parameters with significant effects (*P* < 0.05) that met the assumptions of the test were represented. Finally, a non-parametric analysis of similarities (ANOSIM) with 9999 permutations (free) was conducted after Clarke (1993) to validate the significant differences between interstage microbial fingerprints at WTW F and fingerprints from different SR outlets.

## Results and discussion

### FCM to assess microbial reduction.

**Figure 1.**
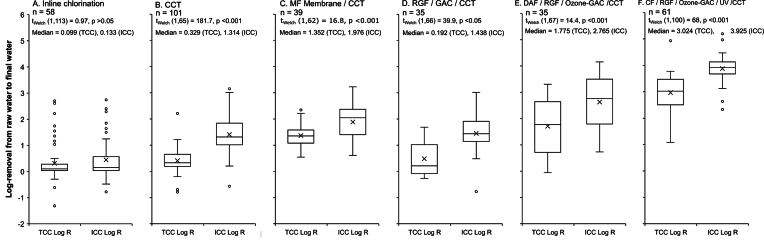
Log reduction of total and intact cells across six strata of WTW (raw/CCT outlet) between April 2019 and March 2020. n is the number of samples for each WTW. Median initial cell number is presented as log_10_ of TCC and ICC. Clarification (CF), CCT; rapid gravity filter (RGF); GAC; microfiltration membrane (MF); GAC.

**Figure 2.**
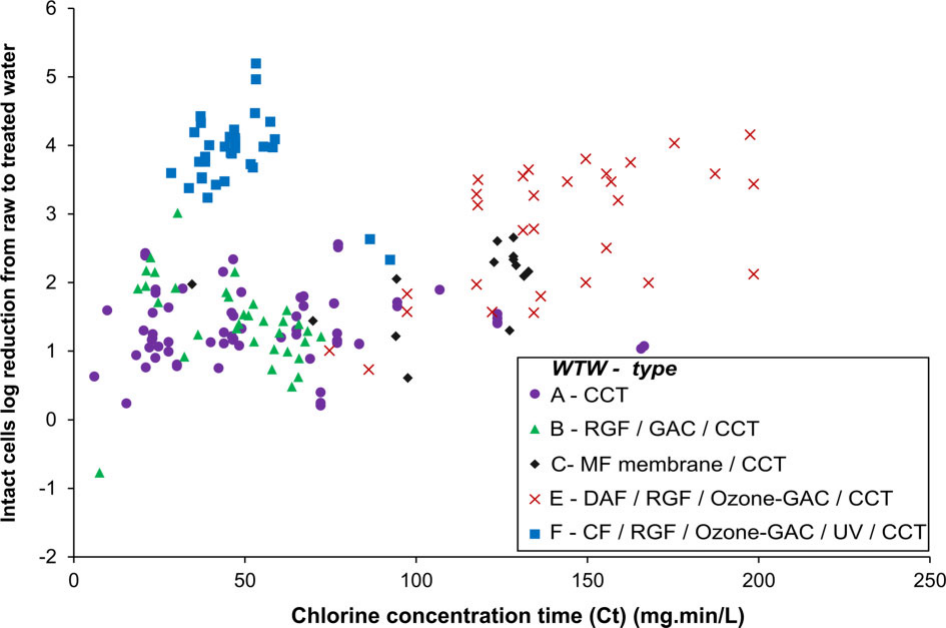
Effect of chlorine CT on Log reduction of intact cells across distinct water treatment trains. This table presents the influence of CT with chlorine on the log reduction of intact cells in various treatment processes including CCT, microfiltration membrane Reactor (MF), GAC, rapid gravity filter (RGF), and GAC.

The results show that the more treatment stages there were in WTWs, the higher the ICC log-reduction. SW treatment showed higher ICC reductions (0.1–4.47 logs) than GW (0.21–3.02 logs), reflecting higher microbial loads and more intensive treatment of SW (Fig. 1). Microbial counts varied from 3.3 × 10³ to 1.3 × 10^7^ TCC/ml in SW and 1.4 × 10² to 2.0 × 10^5^ TCC/ml in GW, highlighting the importance of thorough treatment and disinfection in improving microbial water quality (Fig. 1). WTW A, using basic inline chlorination for GW treatment, had a negligible Ct value and unchanged TCC and ICC counts before and after the process, indicating ineffective cell inactivation. Counts were a low concentration at 4.0 × 10^1^–1.2 × 10^5^ ICC/ml before and 4.0 × 10^1^–1.7 × 10^4^ ICC/ml after, with a <0.09 log reduction (t_Welch_ 0.97, (1 113), *P* > 0.05; Fig. 1A), as chlorine was only used to maintain residual rather than disinfect. Conversely, other WTWs (Fig. 1B–F) significantly reduced TCC and ICC (t_Welch_ in all cases, *P* < 0.05). At Site A, the median viability was 81% at the chlorine tank inlet and barely changed after inline chlorination (80% viable). Other WTWs averaged 65% inactivation, ranging from 62% in WTW C to 70% in WTW E. While upstream processes reduced microbes for CCT disinfection, a positive Ct-ICC reduction correlation (R^2^ = 0.804, *P* < 0.05) emerged, albeit subtler than anticipated, which could result from the varied chlorine susceptibility among drinking water microbial taxa, underscoring FCM's sensitivity of FCM in assessing disinfection and the role of upstream processes in water disinfection (Cheswick et al. 2022, Claveau et al. 2024). FCM data indicated a 0.191 log cell reduction per 10 mg min/l increase in Ct (Fig. 2). CCT concentration time and upstream processes affect cell reduction (Cheswick et al. 2019), yet studies show variable susceptibility among isolates to chlorine levels, as higher chlorine concentrations for shorter periods were more effective than lower concentrations for longer durations; (Mao et al. 2021). However, other factors influence the response of bacteria to different disinfectants. Given the preliminary stresses imposed on microbial populations by the initial WTW processes, the disinfection mechanism—oxidative cell damage from free chlorine and/or ozone—becomes more effective (Fig. 1E). This was attributed to the heightened susceptibility of these pre-stressed cells to protein denaturation, lipid peroxidation, and nucleic acid disruption(Ding et al. 2019). Thus, while preceding treatments either removed the cells or caused changes in the physiology or permeability of the protective cell envelope, the oxidative action of chlorine and ozone ensured thorough disinfection(Dietrich et al. 2007).

**Figure 3.**
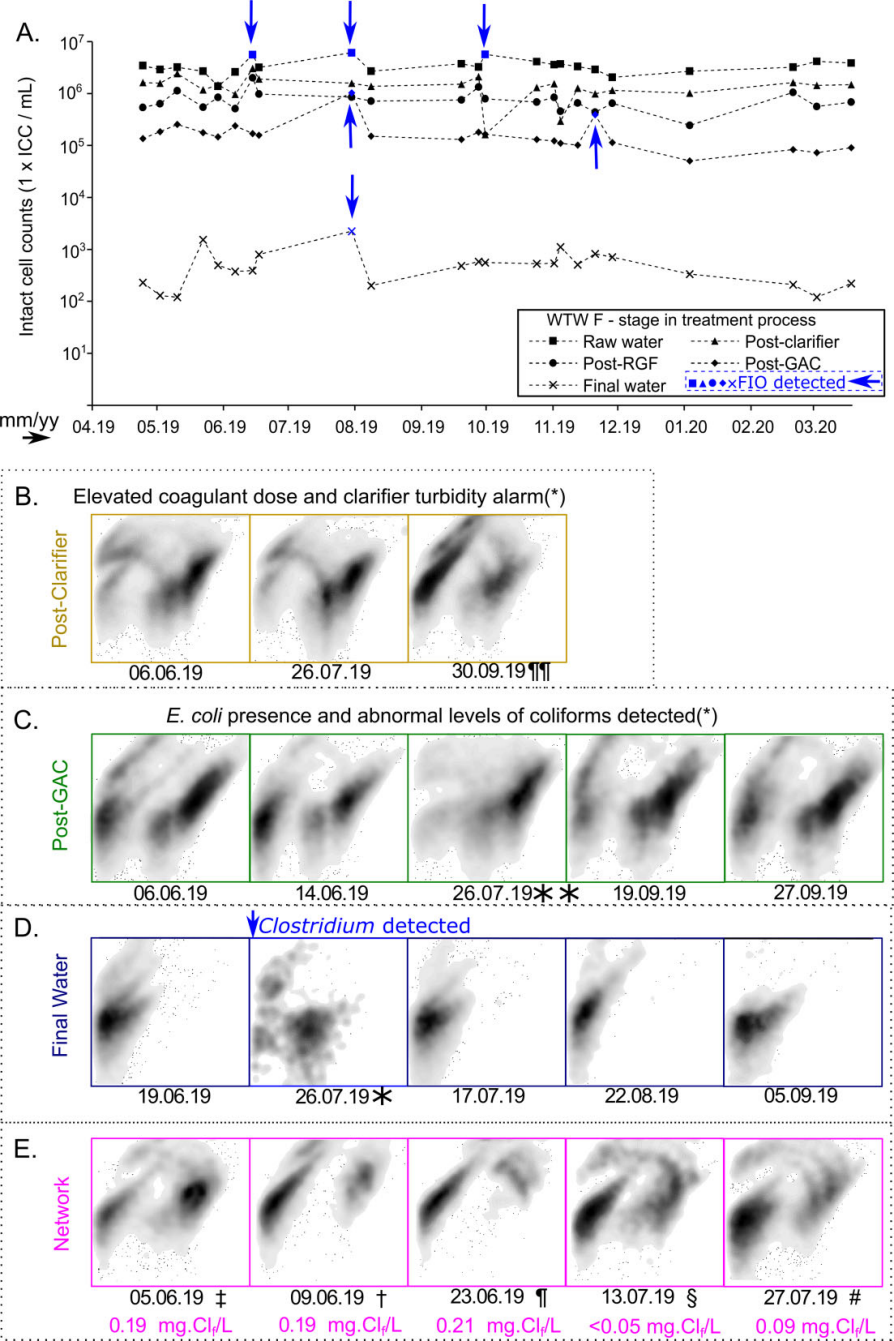
(A) ICC abundance in the different process stages of the WTW. Samples were obtained at Raw and post—CF, RGF, Ozone/GAC, and UV/contact tank. Blue highlighted symbols represent FCM data obtained alongside detection of in process *bacteria faecal* indicator organisms (FIOs) in linked samples. Blue square = *E. coli* > 2420 MPN/100 mL, Blue diamond = *E. coli* > 1 MPN/100 mL, Blue cross = *Clostridium perfringens* > 1 MPN/100 mL. (B–E) are grey scale fluorescent fingerprints with green fluorescence FL-1 channel at 533 nm and the red fluorescence in the FL-3 channel at 670 nm. B. microbial fingerprints linked to a process alarm from the pot-clarified turbidity C. microbial fingerprints linked to abnormal detection of faecal indicator bacteria in the post-GAC samples, (D) fingerprints before, during and post a detection of *C. perfringens*, E. Network samples obtained during periods of normal 0.19 mg/l free chlorine and low < 0.09 mg/l free chlorine (footnotes, †, ‡, §, #, ¶ refer to the components within Fig. 4A). Rapid gravity filter (RGF), GAC, Free chlorine residual (Cl_f_)., ICC. All fingerprints in (B–E) included background.

The largest ICC log reduction occurred across the CCT (2.08–3.35) (Fig. 3A), while coagulation/clarification removed most cells, eliminating 5.7 × 10^6^ ICC/ml (Fig. 1). The median ICC log reduction across the whole process was 1.5 for MF membrane/CCT WTWs, while CF/RGF/ozone-GAC/UV/CCT WTWs showed a total log reduction of 3. The latter, treating SW, had log reductions that ranged between 3.1 and 5.2, averaging 47.3 mg min/l Ct, compared to 0.8–2.2 mg min/l for CCT and RGF/GAC/CCT with similar Ct (Fig. 2). Figure 2 shows that the WTW with UV and ozone achieved higher ICC removal than its CT value, likely due to synergistic cell damage across the two processes. Although the contribution of ozone to log reduction is well documented (Cho et al., 2010; Ramseier et al., 2011), current FCM methods are unable to quantify the mechanisms of UV disinfection accurately. This limitation arises because UV disinfection targets DNA/RNA, and there is an absence of appropriate cellular markers for UV-induced damage that can be detected through conventional FCM techniques (Blyth et al. 2021, Farrell et al. 2018, Wang et al. 2010). As such, we reveal that coagulation/clarification treatment is most effective in reducing cell concentrations, with ozone and UV treatments in WTWs showing possible synergistic effects, with above trend removal efficiencies based on concentration-time values, highlighting the need for further research into UV-disinfection mechanisms and specifically culture-independent approaches for determining its efficacy (Blyth et al. 2021).

### Relating FCM data to coliform detections

Ensuring microbial compliance, gauged by the absence of FIOs in the final delivered water, is of paramount importance to water utilities. The normalised frequency of FIO-positive samples (detections/sampling effort) in GW with inline chlorination was 0.007%, compared to 0.004% for the SW RGF/GAC/CCT process and 0.001% for both SW coagulation-CF/RGF/ozone-GAC/UV/CCT (WTW F) and DAF/RGF/Ozone-GAC/CCT (WTW E) processes. Raw water from surface sources had high FIO detections, with 78% (WTW-E) and 96% (WTW F) detection frequencies in these sources, as expected. However, this was 9.75 to 12 times higher than that in the GW, which had a 0.08% detection rate (WTW A). The SR samples showed negligible FIOs detection (Table 1). The WTW from GW had low final water FIO rates (0.007%), but these were higher than those for SWs (0.001%–0.004%) despite more sampling effort (Table 1). Sites with more FIOs, including GW-fed works with inline chlorination and CCT-only works, showed higher ICC in treated water, unlike complex SW systems with extensive Ct and pretreatment—reducing cells. This implies that what may be considered as ‘low-risk’ GW may need stronger disinfection for compliance and safety, especially when we consider (re)-emerging risk factors such as *Cryptosporidium* and enteric viruses (Singh et al. 2022).

**Table 1.** Summary of E. coli/coliform positive sample detection across six treatment trains.

		# of samples with positive detection of total coliforms and/or *E. coli*			Frequency of samples with positive detection of total coliforms and/or *E. coli*		
WTW	WTW type	RW	FW	SR	RW	FW	SR
A	Inline chlorination	5	1	0	0.08	0.007	0
B	CCT	10	1	0	0.09	0.001	0
C	RGF/GAC/CCT	1	2	0	0.03	0.004	0
D	MF Membrane/CCT	32	0	0	0.42	0	0
E	DAF/RGF/Ozone-GAC/CCT	29	0	0	0.78	0	0
F	coagulation-CF/RGF/ozone-GAC/UV/CCT	52	1	0	0.96	0.001	0

The table provides an in-depth review of both the quantity and frequency of samples testing positive for *E. coli* and coliforms across six different treatment trains examined in this study. The treatment methods include CCT, rapid gravity filter (RGF), GAC, microfiltration membrane (MF), and GAC.

Despite extensive sampling, GW sources show higher FIO rates in the final water compared to SW treatment processes, revealing a challenge in ensuring microbial safety across different water sources. One reason for this can be found in bacterial physiology, which is different in surface and GW sources. For example, bacteria vary in size and shape, typically ranging from 0.5 to 5.0 μm. Exceptions include the genus *Mycoplasma* which has smaller cells (0.2 μm, and is often pathogenic, while bacteria such as *Epulopiscium fishelsoni* are large up to 500 μm. Ultramicrobacteria, under 0.5 μm, are common in environments such as GW (Luef et al. 2015). Their small size improves nutrient uptake via a higher surface-to-volume ratio and may enable them to pass through some water treatment processes that rely on size exclusion mechanisms, such as filters and membranes (Park et al. 2018). Small bacteria are considered to have a small genome (less DNA) and thus do not fluoresce. Current FCM methods struggle to quantify very small and dim fluorescent LNA, which can partition with the background or may be excluded via gating. The ICC log-reduction for MF-membrane WTW was 0.61–2.66, lower than the 0.9–4.9 range found by Cheswick et al., (2019) likely due to the use of finer ultra-filtration membranes (∼0.01 μm) versus the tested MF membranes (0.1–10 μm). This site had low microbial raw water loading (3.34 × 10³-9.22 × 10^4^ ICC/ml; Fig. 3A), with peak coliform detections at FCM concentrations below 1000 ICC/ml, challenging the use of FCM counts at or around the LOD(Cheswick et al. 2019). High-risk waters showed notable cell reduction, but FCM counts did not correlate well with *E. coli* or coliforms. Part of the reason for this is that FCM methods currently applied for drinking water assessment do not distinguish pathogenic bacteria from benign or beneficial bacteria, limiting their use in hygienic water quality evaluations. It was proposed herein that distinct fingerprints, measured via the Bray–Curtis index, might provide additional insights into the *E. coli* and coliform detection intricacies, potentially tied to changes in the ratio between microbial and background components of the fingerprinting data.

In the context of aquatic bacteria, an abnormal FCM fingerprint can indicate changes in bacterial cells due to physiological alterations, disease states, water treatments, or environmental conditions. These changes can manifest as variations in cell size, granularity, internal complexity, or expression of surface markers, altering the typical FCM profile. In addition, changes caused by different species in raw water or biologically active filters are expected to change the fingerprint. Identifying the specific causes of these alterations requires correlating FCM data with environmental and microbiological analyses to understand how these factors affect water quality and aquatic ecosystems. LNA bacteria, with lower fluorescence and SSC, suggest a small size and reduced metabolic activity, which are common in oligotrophic environments such as GW (Hammes et al. 2008, Prest et al. 2013). They are physiologically distinct and have significantly lower ATP levels than HNA cells do (Cheung et al. 2015, Zlatanović et al. 2017). Studies have shown LNA's ecological importance in various ecosystems, including distinct freshwater sub-communities (Proctor et al. 2018). 16S rRNA sequencing of LNA bacteria from freshwater has linked them to the polynucleobacter cluster (Wang et al. 2009). Ultra-small bacteria in GW, especially from the WWE3, OP11, and OD1 phyla, have been identified with features such as minimal genomes and small cell sizes, similar to traditional definitions of LNA bacteria (Luef et al. 2015). Metagenomic analysis and < 0.2-μm filtration revealed these characteristics, while cryo-electron microscopy showed adaptations for nutrient-scarce environments (such as large surface area and cellular packing density). This provides insights into how the bacterial ecology of drinking water can affect FCM fingerprints. However, additional research is required to understand whether ecological shifts act as precursors to poor water quality outcomes (Claveau et al. 2024).

### Cell counts in full scale DWDS

Ensuring microbial quality in DWDS is complex because of factors such as aging infrastructure, the extent of upstream treatment types, and fluctuating disinfectant residual. In addition, the interplay between water age and temperature influences bacterial regrowth and corrosion (Blokker et al. 2016), with temperature significantly impacting water microbiology, as temperature variations impact DWDS integrity and can exacerbate bacterial proliferation and ingress, potentially impacting the effectiveness of water treatment processes (Schleich et al. 2019). Post-treatment, chlorine residual is vital to mitigate risks, especially in areas with aging infrastructure, higher ambient temperatures, long water storage or travel times, and fluctuating source water quality. These conditions increase the risk of bacterial contamination, making chlorine an essential disinfectant for ensuring microbial safety. A few countries (such as Netherlands) can distribute water without chlorine due to ‘advanced,’ consistent water treatment processes and well-maintained, contamination-resistant infrastructure. Studies have utilized FCM for specific processes (Chan et al. 2018) and have characterized microbial community shifts across WTWs (Vaz-Moreira et al. 2013). This research employed FCM along with chlorine concentration, temperature, and distance from the WTW to understand their combined impact on drinking water microbial populations.

**Figure 4.**
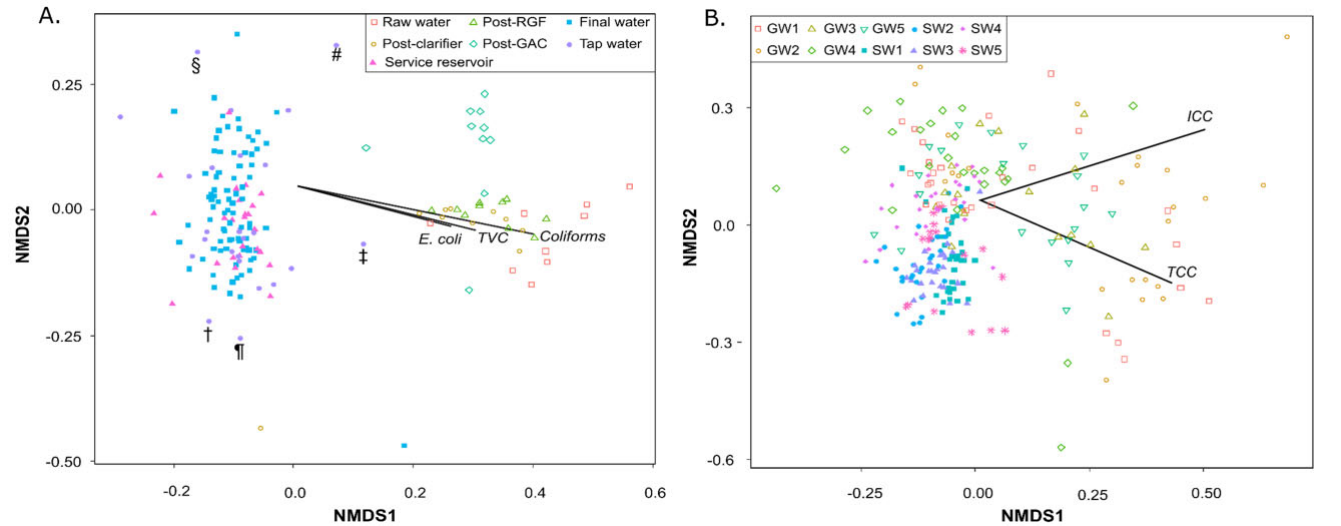
Microbial community profiling using nMDS and CHIC analysis. (A) NMDS ordination plots derived from CHIC analysis of FCM fingerprints, presenting a visual clustering of water samples into three categories based on bacterial communities: i) Pre-disinfection samples, ii) Post-GAC samples, and iii) Post-disinfection samples. Correlation vectors depict the linear relationships between FCM fingerprint and plate count data for heterotrophs, coliforms, and *E. coli*. B. nMDS ordination plots representing FCM fingerprints obtained from ten different contact tank outlets treating water from varying sources within the same water utility. Visual clustering is observed for samples originating from SW and GW. Correlation vectors illustrate the linear associations between the FCM fingerprints and plate count data for heterotrophs, coliforms, and *E. coli*. Data in Fig. 4 (A) has appeared in a different form in Claveau et al. 2024.

**Figure 5.**
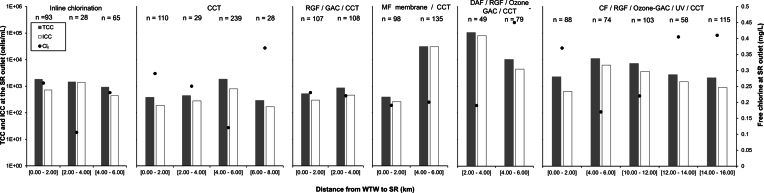
Impact of SR distance on cell counts and residual chlorine concentration. This table depicts the correlation between the distance of the SR from the WTWs and key variables including TCC and ICC at the SR outlet, and residual chlorine concentration. The latter is symbolized with circles in the table.

The weekly variation in raw water revealed three high TCC/ICC spikes, the first (06.06.2019) showing 6.75 log_10_ ICC/ml and >2420 MPN *E. coli*/100 ml. No increase in cell or culture count was noted post-GAC, indicating that ozonation (Vital et al. 2012) and/or GAC filtration (Pinto et al. 2012) provided effective cell removal and process resilience. SRs fed by these WTWs were examined for their distribution/storage impact on water quality. Of the 2107 SR outlet samples, each was categorized into six groups based on preceding WTW trains, and then sub-grouped by WTW-SR distance and the median TCC, ICC, and corresponding residual chlorine analysed (Fig. 5). ICC fluctuated with the WTW-SR distance (Bal Krishna et al. 2013), highlighting a complex interplay between varying source water quality, ICC regrowth, free chlorine, and water age. Despite varying free chlorine (0.1–0.35 mg/l), similar cell counts were observed in GW flowsheets (1.7 × 10^2^–1.4 × 10^3^ ICC/ml), indicating biological stability regardless of distance/water age. Fingerprint analysis indicated two distinct populations: (1) final waters from SW and (2) those from GW, with significant inter- and intra-population differences (R^2^ = 0.3181; *P*-value < 0.001). This variability, seen in Figs. 3E and 4B, could stem from blended supplies, occasional low free chlorine, and notably inconsistent fingerprints in some SW-derived waters, highlighting the variability of these sources.

For SW-based WTW, pretreatment steps influenced microbial population outcomes. For example, the MF membrane/CCT showed a 2 log_10_ cell increase from 0–2 to 4–6 km, showing regrowth in water with free chlorine <0.19 mg/l, while WTW F's more extensive DWDS showed no regrowth (Fig. 5) with similar free chlorine residual. WTW B, E, and F show 2-fold increases of chlorine residual at the farthest points in the network, which was linked to ‘booster’ chlorination. Booster chlorination involves adding chlorine to water within the distribution system to maintain disinfectant levels, ensuring water safety across the network especially in areas far from the treatment plant or in systems with long retention times. FCM has therefore shown that it can be applied to assessments of pipe/SR conditions affecting chlorine decay and cell regrowth (Nescerecka et al. 2018, Nguyen et al. 2012) and inform booster chlorination locations/doses in DWDS (Gibbs and Hayes 1989).

### FCM during microbial events

During one operational event at WTW F, which was recorded during this study, indicated a 49% increase in raw water turbidity, coinciding with a WTW alarm due to turbidity >0.1 NTU in clarified and filtered water, and an increase in HNA within the microbial population in the post-clarified water (Fig. 3B). On another occasion, there was a peak in the raw water ICC with 6.79 log_10_ recorded per mL on 26 July 2019 (Fig. 3A). This event coincided with high numbers of post-GAC cultured cells, showing 2420 MPN *E. coli*/100 ml, along with a visibly different (Fig. 3C) and significantly distinct FCM fingerprint based on the difference between Bray–Curtis scores (Fig. 4A). The final water peak was 3.35 log_10_ ICC/ml was coincidental with statistically different fingerprints (based on Bray-Curtis similarity) and detection of *C. perfringens* detection (>1 CFU/100 ml), which occurred on 26 July 2019 in the final treated water (Fig. 3D). The FCM fingerprint differed notably from samples on 19 June 2019 and 17 July 2019 (Fig. 4A) based on Bray–Curtis similarity. CHIC analysis revealed distinct cytometric fingerprints in WTW F interstage samples, categorizing them into three groups: (1) raw water/post-CF/post-RGF; (2) post-GAC; (3) final water/SR outlet/tap water system (Fig. 4A). An ANOSIM test validated these differences, indicating substantial variance between and within the populations (R-value = 0.4963; *P*-value = 0.0001). The change in microbial quality from CFs, marked by increased HNA content bacteria after a coincidental increase in raw water turbidity, suggests potential inefficiency in the clarification process, possibly leading to elevated levels of intact bacteria in post-clarified water. This suggests the potential for knock-on downstream impacts that could compromise water safety or put added stress on vital processes such as disinfection. In this instance the failure in the WTW, did not coincide with evidence of ‘enhanced growth’ conditions or other indicator of adverse stability of the water—i.e. there is not a lagged increase in counts in the SR or taps following this based on our relatively limited sampling (Fig. 4A). However, interestingly, the FCM fingerprint circa 1 day post the event was visibly similar (Fig. 3E) but statistically dissimilar to other tap water samples (Fig. 4A), which is represented by (#) in each figure. This passage highlights the effectiveness of FCM in drinking water surveillance. Despite no immediate signs of water quality issues post-event at the WTW, FCM detected subtle yet significant changes in the water microbial fingerprint when a user-independent fingerprinting analysis approach was undertaken. Critically, this fingerprinting was undertaken with the background particles included, suggesting that in treated water scenarios with a low residual cell count, this approach has merit. This indicates FCM's value in identifying potential safety issues in water that might not be obvious through standard monitoring, enhancing early detection and response, and raising the possibility of computer-assisted water quality surveillance in drinking water (Sadler et al. 2020).

However, current approaches applied in the water sector rely on binary discrimination to quantify microbial fingerprint differences, noting that HNA bacteria are generally larger and more active organisms (Gasol et al., 1999), whereas LNA bacteria primarily consist of relatively small, less active bacteria in environmental water samples (Wang et al. 2009). Although LNA bacteria are active they can also represent a different physiological state in some bacteria (such as persister cells—a phenotypic state linked to environmental stress) (Hewitt and Nebe-Von-Caron 2004). The genome size (reflected by the fluorescence nucleic acid content) of pathogenic microorganisms have at least a two log difference in genome sizes e.g. *Mycoplasma genitalium* (5.8 × 10^5^ base pairs) (Fraser et al. 1995), *E. coli* O157: H7 (5.44 × 10^6^ base pairs) and the largest prokaryote *Sorangium cellulosum*, which has a genome 1.3 × 10^7^(Han et al. 2013). Diversity within heterogeneous microbial populations is not fully captured by binary data (such as FCM plot gating). Innovations in FCM dyes and equipment may enable water treatment operators to identify the cells of concern within fluorescence fingerprints for a more detailed analysis of bacterial indicators and pathogens.

This study highlights the value of combining interstage FCM monitoring with fluorescent fingerprinting to help operators manage water treatment when high cell concentrations are present. Standard FCM data alone did not enable the link to the process event due to insufficient specificity, as sometimes FCM numbers will increase with no concomitant detection or increase in FIO. This is probably a facet of the superior inactivation of pathogens compared to the background microbial community, transient detections in raw waters, and imperfect nature of the FIO tests (Hassard et al. 2016). However, when paired with fluorescent fingerprinting, it was able to further indicate heightened microbial risk due to water instability due to statistically derived differences in the fingerprint. Further verification and systematization are required to make it a practically useful tool for microbial risk assessment. A retrospective investigation by water company operators revealed that from 23 July 2019, the site had a low ozone residual concentration (<0.01 mg/l for four days, resulting in a low ozone Ct (Supplementary Fig. S1A). Peak cell counts in water reached 6 log_10_ ICC/ml on 26 July 2019. Reduced log reductions were observed during a similar event on 23 November 2019 (Supplementary Fig. S1B). The FIO detection could be linked to the higher chlorination Ct required due to the overall lower disinfection during ozonation and thus more pressure on chlorination (Léziart et al. 2019, Örmeci and Linden 2002). Implementing operational set-points using inline FCM at the final water point or interstage discrete FCM monitoring could provide operators with enhanced reporting to improve situational awareness; (Sadler et al. 2020). As this study demonstrates, FCM facilitates rigorous site-to-site comparisons of bacterial metrics. Further work could explore different approaches to normalization and explore the creation of standardized indices for what represents poor microbial water quality, with the potential for machine learning/artificial intelligence, as shown by, Kyritsakas et al. (2023) who developed an algorithm trained to provide timely interventions by detecting baseline deviations with possible benefits of encouraging enhanced drinking water safety.

In this study, few abnormal fingerprints were characterized around an operational ‘event’ and compared to many fingerprints from operational ‘normal’ conditions. Currently, there is limited evidence that abnormal fingerprints do not occur at other times for other reasons. In addition, it is possible that changes to the bacterial population during ‘normal’ operation could occur for example during source water changes (e.g. blending supply) or seasonality etc. As such, there is a minimal indication of the extent of false positives until further work is conducted. Daily monitoring of one WTW underscores the necessity for more frequent data collection to enhance drinking water surveillance. Additionally, the work presented suggests that detailed microbiological data obtained from FCM fingerprinting presents significant opportunities for improving future and sustainable water quality assessments.

In the network, microbial fingerprinting showed a low residual free chlorine impact on microbial regrowth, with significantly higher ICC in warmer water conditions, and samples differed significantly in terms of their fingerprints, as identified by the CHIC/Bray–Curtis dissimilarity index and distance in the NMDS plot (Fig. 4A). Tap water samples had unique microbial fingerprints positioned at the edges of the WTW F-DWDS cluster, but limited sample numbers may have contributed to these differences (Fig. 4A). Some of these tap waters had low chlorine and distinct FCM fingerprints, suggesting the potential for increased, albeit small, risk of regrowth-linked microbial events (LeChevallier et al. 1996); however, it is important to note that this did not translate into significant regrowth (lagged increase in SR/tap water cell counts Fig. 3A and E) or compliance-linked detections (Table 1) at this WTW. FCM, particularly through fingerprint dissimilarity, can inform proactive network management responses such as booster chlorination or network cleaning. The findings of this study on microbial fingerprinting in tap water, particularly the detection of unique fingerprints and varying chlorine levels, can guide operational decisions regarding water treatment. Specifically, the presence of distinct microbial fingerprints, especially where chlorine levels are low, indicates the need for booster chlorination to maintain adequate disinfectant levels and prevent microbial regrowth. Additionally, identifying unique fingerprints at the edges of distribution system clusters may signal areas requiring targeted network cleaning to remove biofilms or microbial biofilm build-up, ensuring water quality and safety. Thus, FCM's ability to detect these microbial variations may aid informed and effective water treatment surveillance and subsequent management—something endorsed in water safety plan approach. Best practices are needed to tailor or optimise drinking water microbial surveillance, particularly under financial or practical constraints. Instead of rigidly adhering to predefined sampling methods, such as weekly fixed-location discrete sampling, it is crucial for implementers to adjust sampling sites and methods flexibly. This adaptation should consider the demographics of communities served by WTW, focusing on high-risk and vulnerable groups, the specific informational requirements necessary for informed decision making, and the infrastructural characteristics that influence drinking water travel times. These infrastructural features can affect drinking quality and storage conditions, and thus, the regrowth potential of biomarkers.

## Conclusions

This study underscores the importance of FCM monitoring and fingerprinting in providing a more comprehensive understanding of the heterogeneous bacterial communities within drinking water systems than what is offered by traditional culture-based methodologies or analyses of FCM ICC and TCC. During the year of intensive analysis across various WTWs and interstage treatments, several insights were generated.

Importance of chlorine Ct controlling cell counts: the findings reaffirm that chlorine Ct is a decisive factor in achieving significant microbial log reduction values, highlighting the essential role of accurate disinfectant dosing in water treatment protocols.

Identification of operational anomalies via fluorescence fingerprinting: the study demonstrated the value of fluorescence fingerprinting in detecting notable operational disruptions, particularly those related to treatment processes. These disruptions were identified as markedly distinct from standard operational fingerprints using CHIC/nMDS analysis.

Correlation between indicator events and biological stability: the analysis indicated that samples associated with bacterial indicator events, notably from raw, post-GAC, and final water stages, frequently displayed fluorescent fingerprints suggestive of opportunities for bacterial growth, that is, biological instability. This observation underscores the need for user-friendly tools to enable operators to differentiate microbial populations through fingerprint analysis.

Impact of chlorine residue on biological stability in distribution networks: the presence of low chlorine residue was observed to notably alter bacterial fingerprints, indicating potential biological instability within certain network samples. Although no direct correlation with pathogenic bacteria was established, the heightened risk identified underlines the utility of FCM in pre-emptively identifying locations with increased risk levels.

Contrasting GW and SW treatments: the data suggest that, despite the higher ICC, GW-derived WTWs showed a reduced frequency of bacterial water quality compliance events compared to SW systems.

Enhanced FCM analysis: the statistical examination of FCM fingerprints during this study offers insights into the bacteriological quality of water, going beyond the information provided by traditional bacterial monitoring. FCM enables a more accurate assessment of disinfection processes and can expedite the characterization of potential infectious agents for immediate and accurate hygienic risk assessments.

In conclusion, this study provides insights that support calls for a more sophisticated FCM-based approach to microbial water quality assessment and lays the groundwork for subsequent studies in this field.

## Supplementary Material

qvae003_Supplemental_Files

## Data Availability

Data associated with this manuscript is available at: 10.17862/cranfield.rd.23735649.
